# Haptoglobin increases with age in rat hippocampus and modulates Apolipoprotein E mediated cholesterol trafficking in neuroblastoma cell lines

**DOI:** 10.3389/fncel.2014.00212

**Published:** 2014-08-05

**Authors:** Maria Stefania Spagnuolo, Bernardetta Maresca, Maria Pina Mollica, Gina Cavaliere, Carolina Cefaliello, Giovanna Trinchese, Maria Grazia Esposito, Rosaria Scudiero, Marianna Crispino, Paolo Abrescia, Luisa Cigliano

**Affiliations:** ^1^Department of Bio-Agrofood Science, Institute of Animal Production Systems in Mediterranean Environments, National Research CouncilNaples, Italy; ^2^Department of Biology, University of Naples Federico IINaples, Italy

**Keywords:** haptoglobin, apolipoprotein E, cholesterol, aging, human neuroblastoma cell line SH-SY5Y, human astrocytoma cell line U-87 MG, hippocampus, cerebrospinal fluid

## Abstract

Alteration in cholesterol metabolism has been implicated in the pathogenesis of several neurodegenerative disorders. Apolipoprotein E (ApoE) is the major component of brain lipoproteins supporting cholesterol transport. We previously reported that the acute-phase protein Haptoglobin (Hpt) binds ApoE, and influences its function in blood cholesterol homeostasis. Major aim of this study was to investigate whether Hpt influences the mechanisms by which cholesterol is shuttled from astrocytes to neurons. In detail it was studied Hpt effect on ApoE-dependent cholesterol efflux from astrocytes and ApoE-mediated cholesterol incorporation in neurons. We report here that Hpt impairs ApoE-mediated cholesterol uptake in human neuroblastoma cell line SH-SY5Y, and limits the toxicity of a massive concentration of cholesterol for these cells, while it does not affect cholesterol efflux from the human glioblastoma-astrocytoma cell line U-87 MG. As aging is the most important non-genetic risk factor for various neurodegenerative disorders, and our results suggest that Hpt modulates ApoE functions, we evaluated the Hpt and ApoE expression profiles in cerebral cortex and hippocampus of adolescent (2 months), adult (5 and 8 months), and middle-aged (16 months) rats. Hpt mRNA level was higher in hippocampus of 8 and 16 month-old than in 2-month old rats (*p* < 0.05), and Hpt concentration increased with the age from adolescence to middle-age (*p* < 0.001). ApoE concentration, in hippocampus, was higher (*p* < 0.001) in 5 month-old rats compared to 2 month but did not further change with aging. No age-related changes of Hpt (protein and mRNA) were found in the cortex. Our results suggest that aging is associated with changes, particularly in the hippocampus, in the Hpt/ApoE ratio. Age-related changes in the concentration of Hpt were also found in human cerebrospinal fluids. The age-related changes might affect neuronal function and survival in brain, and have important implications in brain pathophysiology.

## Introduction

Cholesterol is a key component of biological membranes, participates in the modulation of their fluidity, and is the precursor of several signaling molecules, including steroid hormones (Tabas, 1997; Simons and Ikonen, 2000). Approximately 25% of the total body cholesterol resides in the brain (Dietschy and Turley, 2001), mostly in the myelin sheath that surrounds the axons, where facilitates the transmission of electrical signals and plays a crucial role in the development and maintenance of neuronal plasticity and function (Pfrieger, 2003). The blood-brain barrier prevents the uptake of this lipid from the circulation (Dietschy and Turley, 2004), hence the majority of cholesterol present in this organ is synthesized *in situ* (Dietschy and Turley, 2001; Pfrieger and Ungerer, 2011). In particular, as intracellular synthesis is down regulated in the mature brain, neurons meet their cholesterol requirements essentially via uptake of cholesterol carried in Apolipoprotein E (ApoE)-containing lipoproteins secreted by glial cells (Pfrieger, 2003). This specific brain lipoprotein transport system very efficiently recycles cholesterol, and mainly depends on the presence of ApoE (Verghese et al., 2011). Astrocytes release cholesterol and ApoE that are assembled with phospholipids into lipoprotein particles, which are similar in size to plasma HDL (Boyles et al., 1985; DeMattos et al., 2001; Pfrieger and Ungerer, 2011). These lipoproteins interact, via ApoE, with the low-density lipoprotein receptor (LDLR) and with the low-density lipoprotein receptor-related protein 1 (LRP1), expressed in neurons (Boyles et al., 1989; Posse de Chaves et al., 2000; Herz, 2009), thus delivering cholesterol for growth, repair, and synaptogenesis (Posse de Chaves et al., 1997; Mauch et al., 2001; Hayashi et al., 2004). In this way, cholesterol is shuttled from astrocytes to neurons (Mauch et al., 2001; Vance and Hayashi, 2010). The maintenance of the correct balance of cholesterol is critical for neuronal function, and any alteration in cholesterol levels may severely affect brain performance. The decay on such performance is one of the hallmarks of aging. In addition, impaired brain cholesterol distribution and metabolism has been pointed to as likely involved in the pathogenesis of Alzheimer's disease (AD), and other neurodegenerative diseases (Solomon et al., 2007; Foley, 2010; Vance, 2012). The precise mechanisms underlying the association between altered cholesterol metabolism and neurodegenerative diseases have not been clarified yet. Since ApoE plays a key role in regulating cholesterol homeostasis in the brain (Vance and Hayashi, 2010), it is conceivable that ligands modulating its functions might have critical effect on brain physiology. In this frame, our group previously reported that Haptoglobin (Hpt), an acute-phase protein of inflammation, binds ApoE thus influencing stimulation of cholesterol esterification by the enzyme lecithin: cholesterol acyltransferase and cholesterol uptake by hepatocytes (Cigliano et al., 2009). Hpt is so far known for its role in Hemoglobin (Hb) binding and transport to the liver (Quaye, 2008), and it was initially identified as a marker of blood-brain barrier dysfunction (Chamoun et al., 2001). Further, some studies pointed out that this protein may be produced in the brain in response to different stress stimuli (Lee et al., 2002; Borsody et al., 2006; Zhao et al., 2009), and increased level of Hpt was found in cerebrospinal fluids (CSF) from patients with AD (Johnson et al., 1992), or other neurodegenerative diseases such as Parkinson' and Huntington's disease (Argüelles et al., 2010; Huang et al., 2011). Hpt colocalizes with amyloid plaques in AD (Powers et al., 1981), inhibits amyloid beta (Aβ) fibril formation *in vitro* (Yerbury et al., 2009), acting as extracellular chaperone, and was suggested as potential biomarker of AD (Thambisetty, 2010). We recently reported that Hpt influences ApoE-dependent 24(S)-hydroxycholesterol esterification (La Marca et al., 2014), a key step for cholesterol removal from the brain. Although the role of Hpt in brain pathophysiology has not been fully investigated to date, the ability of Hpt to bind ApoE might disclose an intriguing scenario related to the modulation of cholesterol metabolism in the brain. The objective of this study was to evaluate whether Hpt influences ApoE-dependent cholesterol efflux from astrocytes and cholesterol incorporation in neurons. Moreover, as no data related to modulation of Hpt expression in aging brain are available so far, we studied the expression profiles of Hpt and ApoE in rat cerebral cortex and hippocampus, regions involved in several cognitive and motor functions, and very vulnerable to the aging process.

## Materials and methods

### Materials

Bovine serum albumin fraction V (BSA), gelatin, lecithin, rabbit anti-human Hpt IgG, mouse anti-β actin IgG, goat anti-rabbit Horseradish Peroxidase-conjugated IgG (GAR-HRP), goat anti-mouse Horseradish Peroxidase-conjugated IgG (GAM-HRP), and MTT [3-(4,5-dimethylthiazol-2-yl)-2,5-diphenyltetrazolium bromide], were purchased from Sigma-Aldrich (St. Louis, MO, USA). Recombinant human ApoE2, ApoE3, and ApoE4 were from PeproTech (London, UK). Goat anti-human ApoE IgG, and rabbit anti-goat Horseradish Peroxidase-conjugated IgG (RAG-HRP) were from Chemicon (Merk Chemicals Limited, Nottingham, UK). Mouse anti-human ApoE IgG was purchased from Santa Cruz Biotechnology (Santa Cruz, CA, USA). The ApoE mimetic peptide ^131^EELRVRLASHLRKLRKLRLL^150^ was from INBIOS s.r.l. (Naples, Italy). Rabbit anti-rat Hpt was from ICL Lab (distributed by Prodotti Gianni, Milano, Italy). The dye reagent for protein titration, enhanced chemiluminescence (ECL) reagents, and the polyvinylidene difluoride (PVDF) membrane were from Bio-Rad (Bio-Rad, Hercules, CA). Polystyrene 96-wells ELISA MaxiSorp plates, with high affinity to proteins with mixed hydrophilic/hydrophobic domains, were purchased from Nunc (Roskilde, Denmark). Kodak Biomax light film, Sephacryl S-200, CNBr-activated Sepharose 4 Fast Flow and Blue Sepharose 6 Fast Flow resins were from GE-Healthcare Life Sciences (Milano, Italy). DMEM and fetal bovine serum (FBS) were from BioWhittaker (Verseviers, Belgium). L-glutamine, Neuroblastoma growth supplement N2, penicillin and streptomycin were from Gibco (Life Technologies Italy, Monza, Italy). Cell culture flasks (25 cm^2^), 96-well cell culture plates, and sterile pipettes of Beckton-Dickinson (Milan, Italy) were used. [1α,2α−^3^H]-Cholesterol (52.5 Ci/mmol) and the liquid scintillation counting cocktail Ultima Gold were obtained from Perkin-Elmer (Boston, MA, USA).

### Purification of Hpt

Hpt was isolated from plasma of healthy subjects (phenotype 1-1) by a multi-step purification procedure, based on a gel filtration with a column of Sephacryl S-200, followed by an affinity chromatography with a column of Blue Sepharose 6 Fast Flow, and finally by a further purification by affinity chromatography using a Sepharose resin coupled to anti-Hpt IgG (Cigliano et al., 2009). Hpt was over 98% pure, as assessed by SDS-PAGE and densitometric analysis of Coomassie-stained bands. The molarity of purified Hpt was determined by measuring the protein concentration (Bradford, 1976) and calculating the molecular weight of the monomer α β as previously described (Cigliano et al., 2003).

### Cell culture

The human neuroblastoma cell line SH-SY5Y and the human glioblastoma-astrocytoma cell line U-87 MG were kindly provided by the Institute of Genetics and Biophysics (CNR, Naples, Italy). Cells (500,000) were seeded in 50 ml tissue culture flasks (25 cm^2^ surface), and grown in DMEM supplemented with 10% FBS, 2 mM L-glutamine, 100 U/ml penicillin, and 100 μg/ml streptomycin (complete medium) at 37°C and under humidified atmosphere of 5% CO2 in air. The medium was changed twice a week, and cells were sub-cultivated when confluent.

SH-SY5Y were differentiated to mature neuronal phenotype by incubation in low-serum medium containing retinoic acid (RA), essentially according to published procedures (Påhlman et al., 1984; Nordin-Andersson et al., 2003). In detail, the complete medium was changed to DMEM containing 0.5% FBS, 2 mM L-glutamine, 100 IU/ml penicillin, 100 μg/ml streptomycin, 1% neuroblastoma growth supplement N2. Then, the cells were seeded into 96 well-plates (at 8000 cells/well density), and allowed to attach for 4 h before adding RA (10 μM final concentration). After further 72 h of culture, the differentiated cells were used for cell-based ELISA, and for cholesterol internalization or toxicity assays.

U-87 MG were seeded into 96 well-plates (at 15,000 cells/well density) in complete medium, and incubated (37°C, 5% CO_2_) for 20 h. After removal of the medium, and washing with DMEM, the cells were used for cell-based ELISA or for cholesterol efflux assays as described below.

### Binding of Hpt to ApoE isoforms

All experiments were carried out with liposome-embedded ApoE, as this apolipoprotein, in brain, is mostly associated with HDL-like particles (Vance and Hayashi, 2010). Liposomes containing ApoE2, or ApoE3, or ApoE4 (ApoE: lecithin = 1:133 molar contribution; namely LipoE2, LipoE3 or LipoE4) were prepared by the cholate dialysis method (Chen and Albers, 1982; Salvatore et al., 2009).

Hpt was diluted to a final concentration of 0.01 mg/ml (0.2 μM) with coating buffer (7 mM Na_2_CO_3_, 17 mM NaHCO_3_, 1.5 mM NaN_3_, pH 9.6). Aliquots (50 μl) of Hpt were then incubated overnight at 4°C into the wells of a microtitre plate. After four washes by T-TBS (130 mM NaCl, 20 mM Tris-HCl, 0.05% Tween 20, pH 7.4) and four washes by high salt TBS (500 mM NaCl in 20 mM Tris-HCl at pH 7.4), for removing the excess of Hpt, the wells were blocked with PBS containing 1% BSA and 1% gelatin (1 h, 37°C). The wells were extensively washed, and then incubated (2 h, 37°C) with different amounts (0.005, 0.01, 0.025, 0.05, 0.1, 0.3, or 0.7 μM in TBS) of each ApoE isoform (LipoE2, LipoE3, or LipoE4). The amount of apolipoprotein bound to Hpt was measured by incubating the wells with 60 μl of goat anti-ApoE IgG (1: 2500 dilution in T-TBS containing 0.25% BSA; 1 h, 37°C), followed by 70 μl of RAG-HRP IgG (1: 7000 dilution; 1 h, 37°C). Color development was measured at 492 nm. Absorbance values were converted to the percentage of the value obtained with 0.7 μM Apolipoprotein (assumed as 100% of ApoE binding to Hpt). Data were analyzed by a non-linear regression fit algorithm by GraphPad Prism v 5.01, in order to obtain the dissociation constant (Kd) of each ApoE isoform for Hpt.

### ApoE binding to SH-SY5Y and U-87MG by ELISA

The effect of Hpt addition on ApoE binding to cells was evaluated on both differentiated SH-SY5Y and U-87 MG. As described above, SH-SY and U-87MG were seeded into a 96-well plate (8000 and 15,000 cells/well, respectively), and cultured for 72 h in RA-supplemented medium or for 20 h in complete medium respectively. After removal of the culture medium, the cells were washed with DMEM, and then fixed by incubation (30 min, 4°C) with 0.3% glutaraldheyde in PBS. After removing glutaraldheyde, the wells were gentle washed with PBS, and finally blocked with PBS containing 1% BSA and 1% gelatin (overnight, 4°C). After extensive washing, the wells were incubated (2 h, 37°C) with aliquots (55 μ l) from mixtures containing 0.4 μ M LipoE3 and different concentrations of Hpt (0, 0.04, 0.1, 0.4, 1.6, 4, or 8 μ M). The amount of LipoE bound to cells was measured by treatment with mouse anti-ApoE IgG (1: 3000 dilution in PBS; 1 h, 37°C), followed by GAM-HRP IgG (1: 20000 dilution in PBS; 1 h, 37°C), and color development at 492 nm. Absorbance values were converted to the percent of the value obtained in the absence of Hpt (assumed as 100% of LipoE binding).

### Cholesterol efflux assay

Cellular cholesterol efflux from astrocytes was measured essentially according to Kim et al. (2007). In detail, U-87 MG were cultured (96 well-plates; 15,000 cells/well) in complete medium for 20 h. After medium removal, cells were rinsed with DMEM, and labeled by incubation (20 h, 37°C) with [1α,2α−^3^H]-Cholesterol (52.5 Ci/mmol; 0.026 μCi/well) in DMEM containing 0.5% FBS, 100 IU penicillin/ml, 100 μg streptomycin/ml. The medium was removed, cells were rinsed twice in DMEM and then incubated in serum-free DMEM containing 0.2% BSA, 100 IU/ml penicillin, 100 μg/ml streptomycin (BSA-supplemented DMEM), 0.15 μM LipoE (ApoE: lecithin = 1:133 molar contribution), as cholesterol acceptor, and different amounts of Hpt (0, 0.5, 1.5, 3, or 5 μM). Cells incubated in BSA-supplemented DMEM, or in BSA-supplemented DMEM containing different Hpt concentrations were used as controls. Media samples were collected after 5 h, and cleared of any cellular debris by centrifugation at 400 g for 5 min. The cells were extensively washed with DMEM, lysed with 0.1 M NaOH, and finally centrifuged at 12000 g for 30 min. Aliquots (70 μl) of supernatants and lysates were then analyzed by scintillation counting, and cholesterol effluxed to the medium was calculated as a percentage of total radioactivity in the cell lysates and medium. Experiments were routinely performed in triplicate and repeated three times.

### Cholesterol incorporation assay

The ApoE-dependent internalization of cholesterol into differentiated SH-SY5Y was carried out essentially according to published procedures (Garcia et al., 1996; Cigliano et al., 2009). In detail, cells were seeded in low-serum medium (8000 cells per well) into 96-well plate, as above described, and cultured for 72 h in presence of RA. After medium removal, cells were rinsed with DMEM, and incubated (3 h, 37°C) in BSA-supplemented DMEM containing labeled proteoliposome (ApoE final concentration 10 nM; cholesterol final concentration 15 nM), unlabeled liposome (cholesterol final concentration 80 nM) and different amounts of Hpt (0, 0.25, 0.5, or 1.0 μM). In control experiments, Hb (2 μM) or HSA (5 μM) was added to the culture medium to displace Hpt from proteoliposomes, or to verify the specificity of Hpt effect, respectively.

The specific effect of Hpt interaction with ApoE was further confirmed by incubating differentiated SH-SY5Y (3 h, 37°C) in BSA-supplemented DMEM containing labeled proteoliposome (ApoE final concentration 10 nM; cholesterol final concentration 15 nM), unlabeled liposome (cholesterol final concentration 80 nM), different amounts of Hpt (0, 0.5, or 1.0 μM), and different amounts (0, 4, or 8 μM) of a peptide mimicking the ApoE sequence (^131^EELRVRLASHLRKLRKLRLL^150^; Salvatore et al., 2009) involved in the binding with Hpt. A further control was carried out by incubating cells in the presence of labeled proteoliposome, unlabeled liposome, 1.0 μM Hpt and 7 μg/ml monoclonal anti-human Hpt IgG (Sigma-Aldrich, St. Louis, MO, USA). At the end of incubation, media samples were collected, and cleared of any cellular debris by centrifugation at 400 g for 5 min. The cells were extensively washed with DMEM, lysed with 0.1 M NaOH, and finally centrifuged at 12,000 g for 30 min. Aliquots (70 μl) of supernatants and lysates were then analyzed by scintillation counting, and protein concentration in cell lysates was measured by Bradford assay (Bradford, 1976). The amount of cholesterol internalized was calculated as d.p.m in lysates per mg cell protein. Experiments were routinely performed in triplicate and repeated three times.

Liposomes containing ApoE and lipids were prepared by the cholate dialysis procedure (Chen and Albers, 1982), but with an ApoE/lecithin/cholesterol molar ratio of 1: 100: 2 (Matz and Jonas, 1982). [1α,2α−^3^H]-Cholesterol (specific activity 102.4 x10^6^ dpm × nmol^−1^) in proteoliposome containing 10 μM ApoE was used. Unlabeled liposome, prepared without apolipoprotein, was used to evaluate non-apolipoprotein-mediated uptake of cholesterol. In detail, non-specific internalization was determined in the presence of a 5-fold excess of the unlabeled cholesterol, and represented about 50% of the total internalization.

### Analysis of cholesterol effect on neurons viability

The effect of cholesterol on survival of differentiated SH-SY5Y was evaluated using cholesterol embedded into liposome (cholesterol/lecithin molar ratio 1/50) prepared by the cholate dialysis method (Chen and Albers, 1982). Free ApoE was used for promoting cholesterol incorporation.

SH-SY5Y were seeded in low-serum medium (8000 cells per well) into 96-well plate, as above described, and cultured for 72 h in presence of RA. After medium removal, cells were rinsed with DMEM, and then incubated (20 h, 37°C) in BSA-supplemented DMEM containing free ApoE (final concentration 10 nM), and different amounts of cholesterol (0, 1, 5, 15, or 30 μM final concentration). The medium was then removed and cell survival was evaluated by MTT assay. In detail, 100 μl of MTT (0.5 mg/ml in DMEM without Phenol red) were added to each well and the incubation was carried out for 3 h (37°C). One hundred micro liters of a solution containing 0.1 M HCl in isopropanol were then added to each well, and, after 30 min, the absorbance at 595 nm was measured with a Bio-Rad 3350 microplate reader. The data were expressed as viability percentage, assuming the absorbance value from cells cultured in BSA-supplemented DMEM, containing 10 nM ApoE, as 100%.

The effect of Hpt on cholesterol-mediated cytotoxicity was evaluated by incubating (20 h, 37°C) differentiated SH-SY5Y in BSA-supplemented DMEM containing free ApoE (final concentration 10 nM), cholesterol (15 μM final concentration), and different amounts of Hpt (0, 0.005, 0.01, or 0.05 μM final concentration). Cell viability was then measured by MTT reduction assay, as described above, and data were expressed as cell survival percentage, assuming the absorbance value from cells cultured without cholesterol as 100%.

### Animal and experimental design

Male Wistar rats (Charles River, Calco, Como, Italy) of 2 months of age (adolescent), with the same starting body weight (160 ± 10 g), were individually caged in a temperature-controlled room (23 ± 1°C) with a 12-h light/12-h dark cycle. Animals were housed in the Animal Care Facility at the Department of Biology, with ad libitum access to water and to a standard diet (Mucedola 4RF21; Settimo Milanese, Milan, Italy) up to 5 (social maturity), 8 (adulthood), or 16 (middle-age) months. The animals of each group (*N* = 8) were anesthetized by chloral hydrate (40 mg/100 g body wt) and killed by decapitation. The brains were quickly removed and the cerebral cortex and hippocampus were dissected on ice. Samples of each brain region were snap frozen in liquid nitrogen immediately and stored at −80°C for subsequent RNA and protein isolation.

The protocols for animal care and use were approved by the “Comitato etico-scientifico per la sperimentazione animale” of the University “Federico II” of Naples. All experimental animal procedures were carried out in compliance with national guidelines for the care and use of research animals (D.L.116/92, implementation of EEC directive 609/86). All efforts were made to minimize animal suffering and to reduce the number of animals used.

### Real-time PCR analysis

Total RNA was extracted according to the TRI-Reagent (Sigma Aldrich) protocol. The concentration and purity of RNA samples were determined by UV absorbance spectrophotometry; RNA integrity was checked on 1.2% agarose gel electrophoresis. First-strand cDNA was synthesized from each total RNA (1 μg) using the QuantiTect reverse transcription kit (Qiagen). The Real Time PCR reactions were carried out in quadruplicate in an Applied Biosystems 7500 Real Time System by using the Power SYBR Green Master Mix PCR (Applied Biosystems). Each SYBR Green reaction (20 μl total volume) contained 2 μl of 1:1 diluted cDNA as template. For internal standard control, the expression of β-actin gene was quantified. Primer sequences were designed using Primer Express software (Applied Biosystems). *ApoE* primers were designed on the exon junction 82/83 (forward primer) on template NM138828.2 (forward primer, 5′-GGTCCCATTGCTGACAGGATGCC-3′; reverse primer, 5′-AGAAAGCGTCTGCACCCAGCG-3′); *Hpt* primers were designed on the exon junction 278/279 (forward primer) on template NM012582.2 (forward primer, 5′-TGAGGCAGTGTGTGGGAAGCCC-3′; reverse primer, 5′-TGTGGCCCCAGTGGTGAGTCC-3′); *β-actin* primers were designed on the exon junction 75/76 (forward primer) on template NM031144.2 (forward primer, 5′-ACCCGCCACCAGTTCGCCAT-3′; reverse primer, 5′-CGGCCCACGATGGAGGGGAA-3′). Amplicons were 128–136 bp long. The PCR was performed under the following conditions: holding stage of 95°C per 10′; cycling tage (45 cycles): 95°C × 10 s–60°C × 10 s–72°C × 10 s; melting stage: 95°C × 5 s–65°C × 1 m–95°C × 30 s–40°C × 30 s.

PCR amplification efficiencies were determined for each gene and ΔΔ CT relative quantification was done using β-actin gene expression as internal control to normalize the results.

### Electrophoresis and western blotting

Proteins were extracted from hippocampus and cortex by homogenizing frozen tissues (−80°C) in 10 volumes (w/v) of cold RIPA buffer (150 mM NaCl, 50 mM Tris-HCl, 1% NP-40, 0.5% sodium deoxycholate, pH 8) containing Tissue Protease Inhibitor Cocktail (Sigma-Aldrich, 1:1000, v/v). Homogenates were then centrifuged (14,000 g, 45 min, 4°C) and protein concentration of supernatants was measured according to a published procedure (Bradford, 1976).

Brain homogenates were fractionated by electrophoresis in denaturing and reducing condition on 12.5% polyacrylamide gel, as previously reported (Porta et al., 1999). In detail, samples containing 35 or 50 μg of proteins were analyzed for detecting Hpt in cortex or hippocampus respectively, while samples containing 60 μg of proteins were analyzed for detecting ApoE. After electrophoresis, proteins were transferred onto PVDF membrane (1 h, under electric field), the membrane was rinsed in T-TBS, and then blocked with T-TBS containing 5% non-fat milk (overnight, 4°C). The membrane was then incubated (1 h, 37°C) with rabbit anti-rat Hpt (1: 500 dilution in T-TBS containing 0.25% non-fat milk) followed by GAR-HRP IgG (1: 500 dilution; 1 h, 37°C), or with goat anti-ApoE IgG (1: 1000 dilution in T-TBS containing 0.25% non-fat milk; 1 h, 37°C) followed by RAG-HRP IgG (1: 3500 dilution;1 h, 37°C). The immunocomplexes were detected by the ECL detection system, using luminol as substrate, according to the manufacturer protocol. Quantitative densitometry of ApoE or Hpt was then carried out by analyzing the digital images of membranes by the Gel-Pro Analyzer software (Media Cybernetics, Silver Spring, MA). Band intensities were calculated as Integrated Optical Density (IOD).

After the first detection (Hpt or ApoE), the membrane was extensively washed with T-TBS and then submerged in stripping buffer (100 mM β-mercaptoethanol, 2% SDS, 62.5 mM Tris-HCl, pH 6.7; 45 min, 50°C) for reprobing with anti-β-actin. After washing with T-TBS, the membrane was incubated (1 h, 37°C) with mouse anti- β-actin IgG (1:2000 dilution), followed by GAM-HRP IgG (1:10,000 dilution; 1 h, 37°C). The immunocomplexes were detected by the ECL detection system, and densitometric analysis of the signal was carried out as above. Hpt and ApoE concentrations, in hippocampus and cortex, were quantified after normalization with β-actin, and results were expressed as arbitrary units.

### Analysis of Hpt and ApoE in human CSF

CSF from younger healthy subjects (*N* = 13; 55–70 years) and older healthy subjects (*N* = 12; 71–83 years), were provided by P. Bongioanni (Pisa, Italy). The study conforms to The Code of Ethics of the World Medical Association (Declaration of Helsinki), and it was approved by the Ethics Committee of the University of Naples Federico II.

ApoE and Hpt concentration in individual samples was measured by ELISA. Samples were diluted (1:100–1:800) with coating buffer, and incubated in the wells of a microtitre plate (Immuno MaxiSorp; overnight, 4°C). After four washes by T-TBS and four washes by high salt TBS, the wells were blocked with TBS containing 0.5% BSA (1 h, 37°C). After washing, the wells were incubated (1 h, 37°C) with 60 μl of goat anti-ApoE IgG (1: 3000 dilution in T-TBS containing 0.25% BSA) followed by 60 μl of RAG-HRP IgG (1:14000 dilution) for immunodetection of ApoE, or with 60 μl of rabbit anti-Hpt IgG (1:1500 dilution) followed by GAR-HRP IgG (1:8000 dilution) for Hpt detection. Peroxidase-catalyzed color development from *o*-phenylenediamine was measured at 492 nm.

### Statistical analysis

In all experiments samples were processed in triplicate, and data were expressed as mean value ± s.e.m. The program “GraphPad Prism 5.01” (GraphPad Software, San Diego, CA) was used to perform regression analysis, Student's *t*-test, for comparing two groups of data, and One-Way ANOVA, followed by Tukey's test, for multiple group comparisons. *P* < 0.05 was set as indicating significance.

## Results

### Binding of Hpt to ApoE isoforms

**Figure 1:**
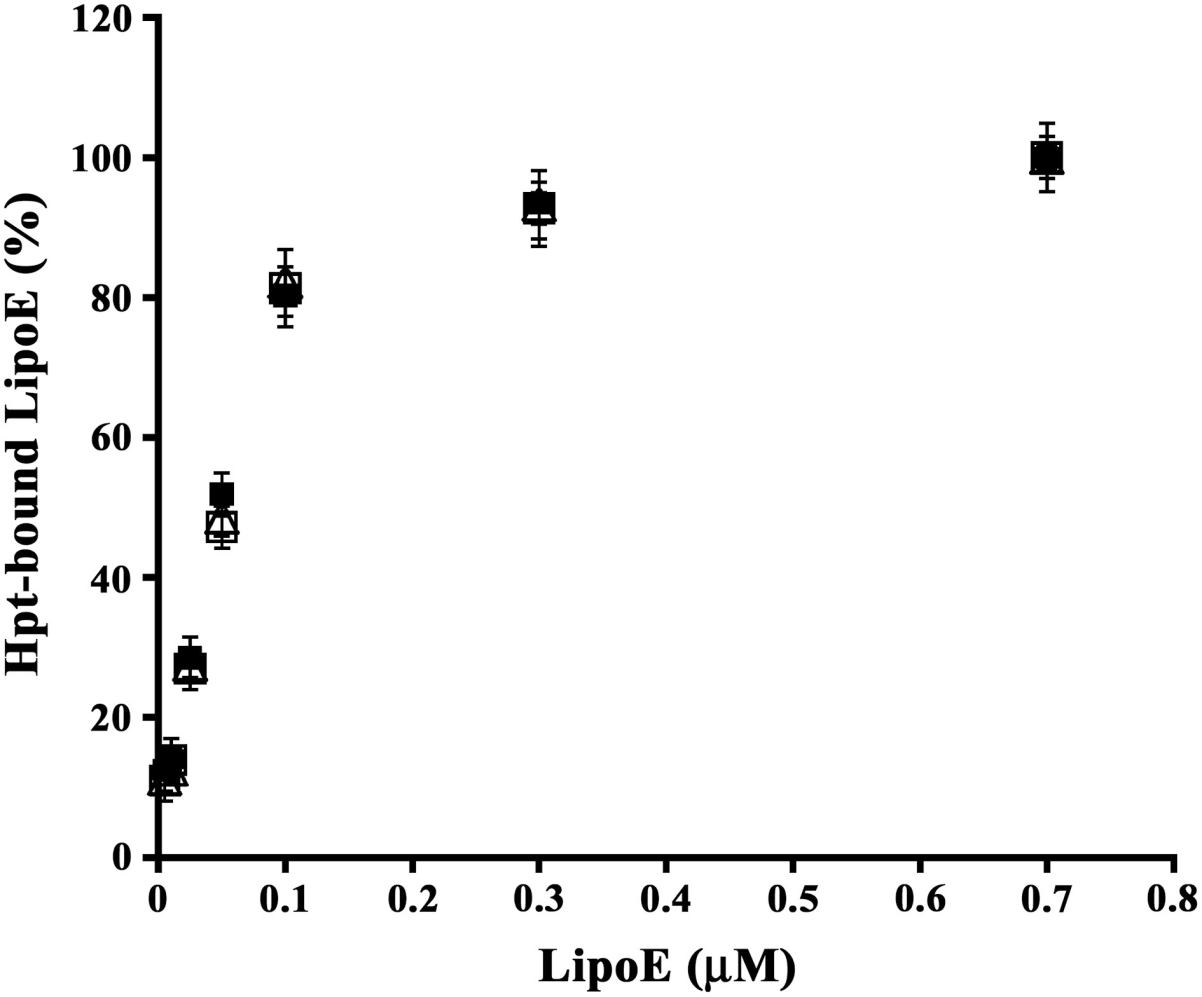
**Binding of Hpt to ApoE isoforms**. Aliquots of 0.2 μM Hpt were loaded into the wells of a microtitre plate, and then incubated with different concentrations (0.005–0.7 μM) of LipoE2 (open triangles), or LipoE3 (open squares) or LipoE4 (solid squares). The amount of apolipoprotein bound to Hpt was detected by goat anti-ApoE and RAG-HRP, measured as absorbance at 492 nm, and reported as percentage of the value obtained with 0.7 μM protein (assumed as 100% of ApoE binding to Hpt). Samples were processed in triplicate. Data were expressed as mean ± s.e.m. vs. μmolar concentration.

As functional differences among the three ApoE isoforms were reported (Castellano et al., 2011; Hashimoto et al., 2012), we firstly investigated whether the isoforms also differ for their ability of binding Hpt. As shown in Figure 1, the amount of ApoE bound to Hpt immobilized into the wells increased with the apolipoprotein concentration in the incubation medium. Kd of each LipoE isoform was calculated as described in Material and Methods, and we found that the three isoforms bind Hpt with similar affinity (Kd LipoE2, 58.24 ± 11.78 nM; Kd LipoE3, 59.58 ± 11.71 nM; Kd LipoE4, 54.88 ± 9.13 nM).

### Hpt influence on ApoE binding to cells

**Figure 2:**
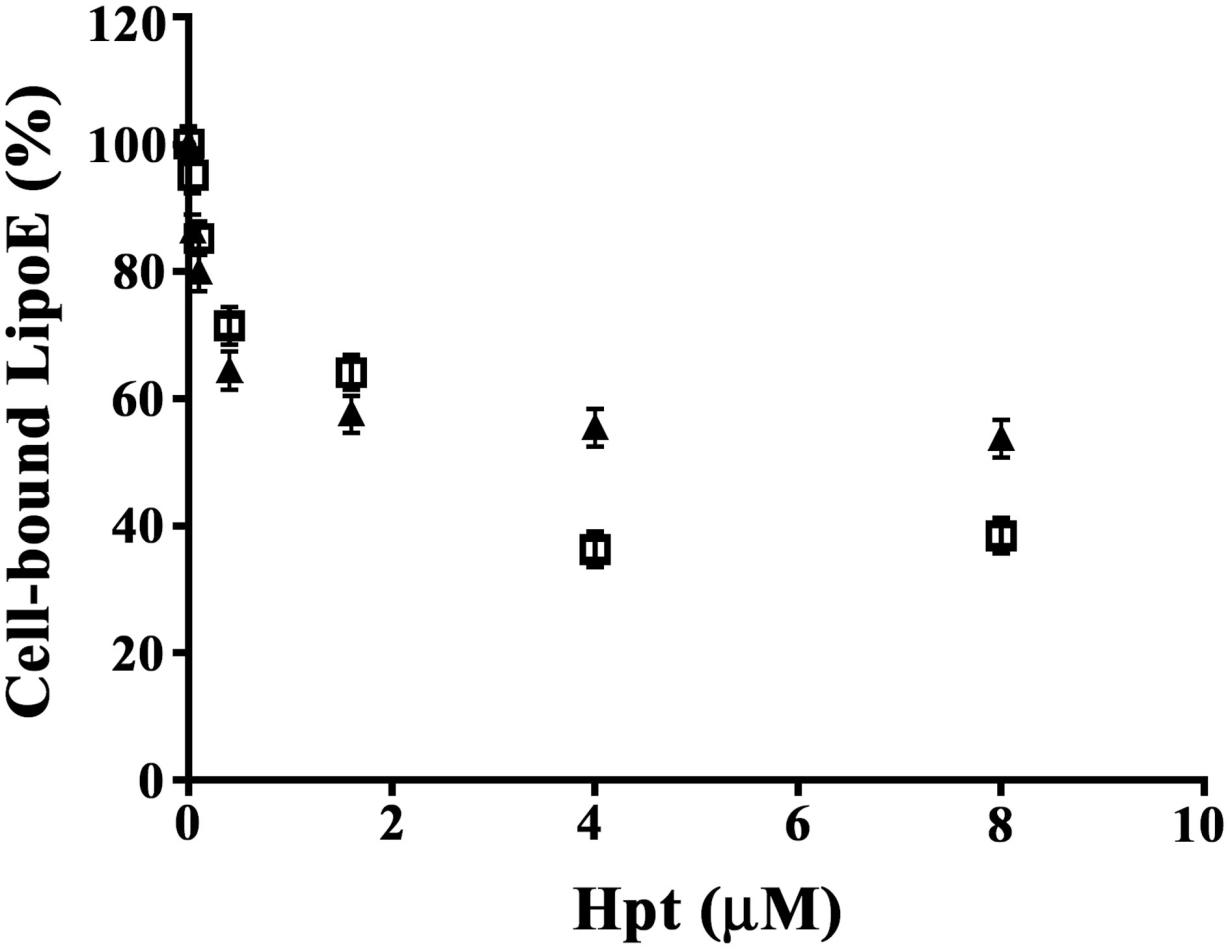
**Hpt influence on ApoE binding to cells**. Differentiated SH-SY5Y (8000 cells; full triangles) or U87-MG (15,000; open squares) were fixed into the wells of a 96-well plate and then incubated with mixtures containing 0.4 μM LipoE3 and different concentrations of Hpt (0–8 μM). The amount of LipoE bound to cells was measured by incubation with mouse anti-ApoE IgG, followed by GAM-HRP and color development at 492 nm. Data are reported as percent of the value obtained by incubation in the absence of Hpt (assumed as 100% of LipoE binding), and expressed as mean ± s.e.m.

As cholesterol uptake by neurons and glial cells is mediated by the binding of ApoE to specific cell surface receptors (Pfrieger and Ungerer, 2011), we investigated whether Hpt, due to its interaction with ApoE, displaces the apolipoprotein from binding to neurons and astrocytes cell lines. Differentiated SH-SY5Y or astrocytes were fixed into the wells of a 96-well plate, and then incubated with mixtures containing 0.4 μM LipoE3 and different concentrations of Hpt 1-1 (0–8 μM). As shown in Figure 2, LipoE binding to cells decreased with the increasing of Hpt concentration in the incubation mixture. Indeed, Hpt significantly reduced (*p* < 0.05) the binding of LipoE3 to both cell types, when used at concentration higher than 0.04 μM. In particular, LipoE binding to SH-SY5Y and astrocytes was reduced of 20 and 15% respectively (*p* < 0.03) by 0.1 μM Hpt. Further, the binding to neurons dropped down to 59 and 57% in presence of 0.4 and 1.6 μM Hpt, respectively (*p* < 0.001), while the binding to astrocytes was reduced to 71 and 64% by the same Hpt concentrations (*p* < 0.01). LipoE binding to SH-SY5Y was halved (*p* < 0.001) by 4 and 8 μM Hpt (presumed 10 and 20-fold excess over ApoE in the mixture), while the binding to astrocytes was reduced to about 35% (*p* < 0.001) by the same concentrations. These results suggest that Hpt interferes with ApoE binding to both neuron and astrocyte cell lines, and this effect is more pronounced when the Hpt/ApoE molar ratio increases.

### Hpt effect on ApoE-dependent cholesterol efflux from astrocytes

**Figure 3:**
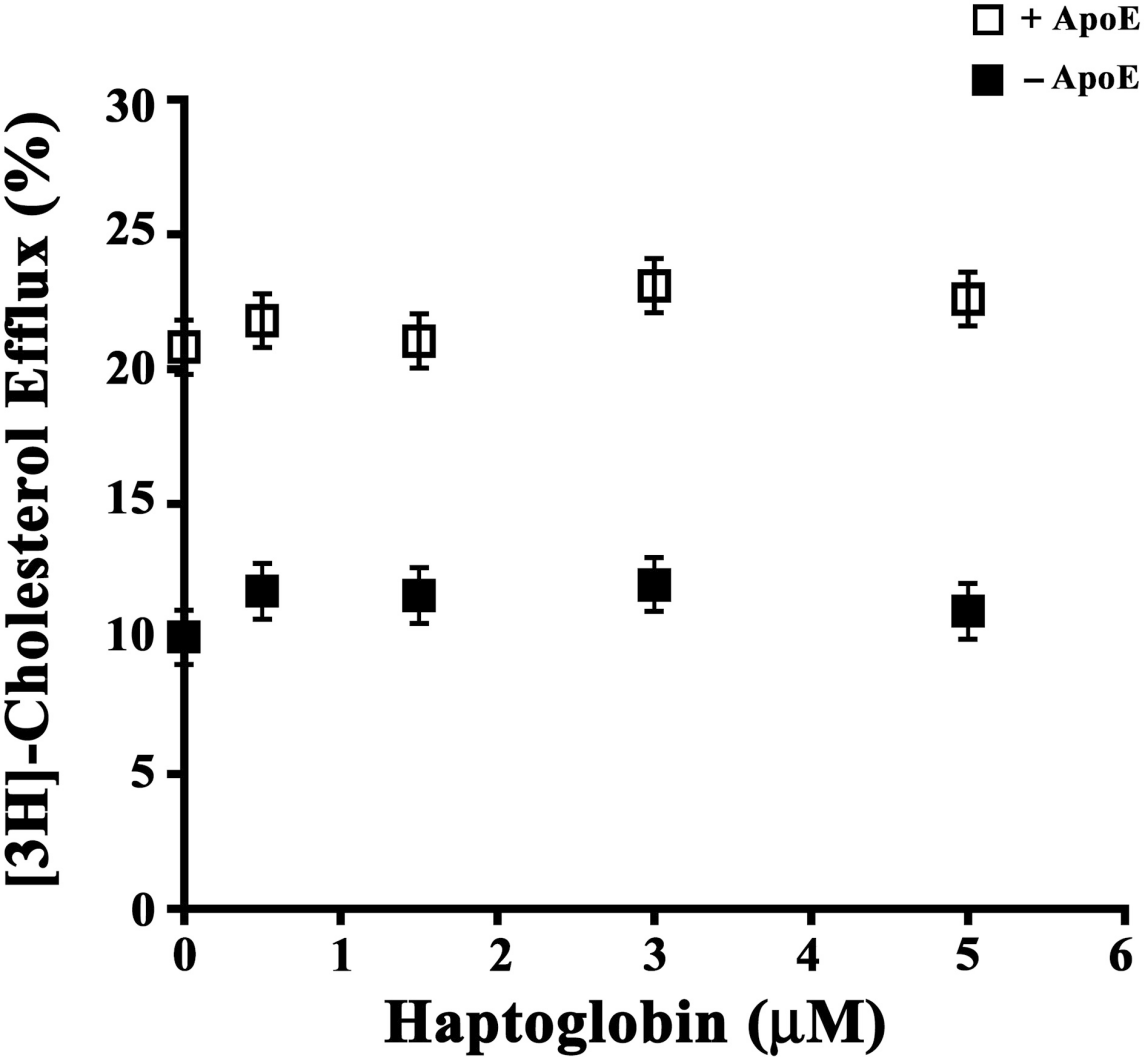
**Hpt effect on ApoE-dependent cholesterol efflux**. U87-MG were seeded into 96-well plate (15,000 cells per well) and cultured in complete medium for 20 h. After medium removal, cells were rinsed with DMEM and labeled by incubation (20 h) with [^3^H]-Cholesterol (0.026 μCi/well) in DMEM containing 0.5% FBS. After medium removal, cells were rinsed again, and incubated (5 h) in serum-free DMEM containing 0.2% BSA in presence (open squares) or absence (full squares) of 0.15 μM LipoE and different amounts of Hpt (0–5 μM). Aliquots (70 μl) of supernatants and lysates were then analyzed by scintillation counting, and cholesterol effluxed to the medium was calculated as percentage of total radioactivity in the cell lysates and medium. Data were expressed as mean ± s.e.m.

In order to evaluate Hpt effect on ApoE mediated cholesterol efflux from astrocytes, cells were labeled with [^3^H]cholesterol (0.52 μCi/ml) for 20 h, and then incubated (5 h) in serum-free DMEM containing 0.15 μM LipoE and different amounts of Hpt (0–5 μM). We found that LipoE significantly stimulated cholesterol efflux from astrocytes (*p* < 0.0001; Figure 3), according with previous data (Kim et al., 2007), and this effect was not impaired in the presence of Hpt, even at the highest protein concentration used (presumed 33-fold excess over LipoE in the mixture). These results suggest that Hpt binding to ApoE does not interfere with cholesterol efflux from astrocytes, and that ApoE retains the ability to stimulate this process also when the Hpt/ApoE molar ratio increases. This might be due to differences in the ApoE sequences involved in the interaction with Hpt and with ABC transporter. Indeed we previously found that Hpt engages the N-terminal region of the apolipoprotein (Salvatore et al., 2009), while the binding of ApoE to the ABC transporters is mediated by the C-terminal region (Vedhachalam et al., 2007).

### Hpt effect on the apoe-mediated cholesterol incorporation

In order to investigate whether Hpt, interacting with ApoE, affects cholesterol internalization by neurons, differentiated SH-SY5Y were incubated (3 h, 37°C) with labeled proteoliposome (ApoE final concentration 10 nM; cholesterol final concentration 15 nM), and different amounts of Hpt (0–1 μM).

**Figure 4:**
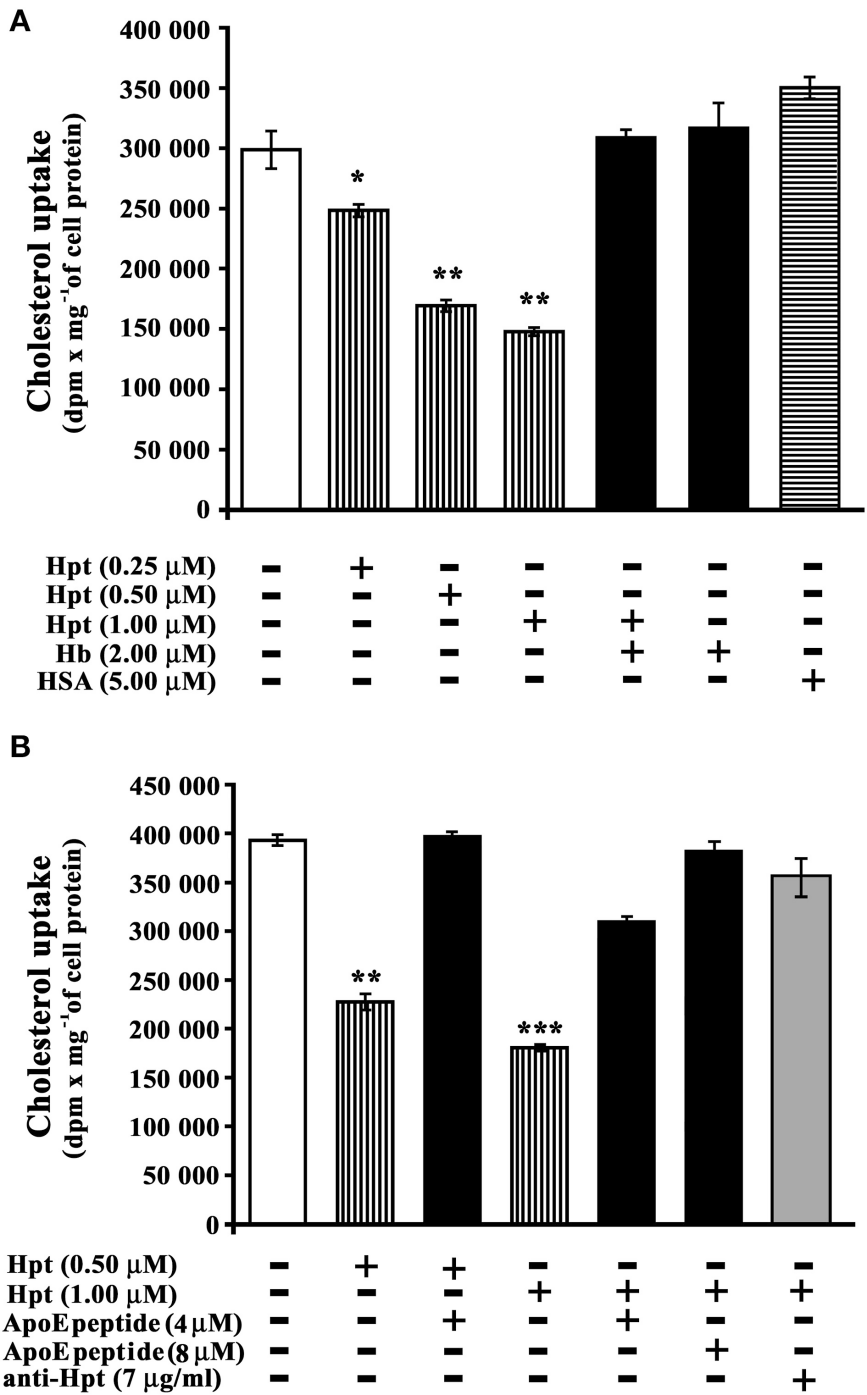
**Hpt effect on ApoE-mediated cholesterol incorporation. (A)** Differentiated SHSY5Y were incubated into 96-well plate (3 h; 8 × 10^3^ cells per well) with proteoliposome suspension containing [^3^H]-cholesterol, phosphatidylcholine, and 10 nM ApoE. The assay was performed in the absence (open bar, control) or presence of different amounts of Hpt (0.25, 0.5 or 1 μM; bars with vertical lines). Control experiments were also carried out in presence of Hb (2 μM; full bar) or HSA (5 μM, bar with horizontal lines). After incubation, the cells were lysed for measurement of their radioactivity and protein concentration. The amount of cholesterol internalized was measured as dpm per mg of cell protein. Significant differences from control are indicated (^*^p < 0.05; ^**^p < 0.001). The samples were analyzed in triplicate, and the data are expressed as means ± s.e.m. **(B)** Differentiated SHSY5Y were incubated into 96-well plate (3 h; 8 × 10^3^ cells per well) with proteoliposome suspension containing [^3^H]-cholesterol, phosphatidylcholine, and 10 nM ApoE. The assay was performed in the absence (open bar, control) or presence of different amounts of Hpt (0.5 or 1 μM; bars with vertical lines). Control experiments were carried out in presence of 0.5 μM Hpt and 4 μM ApoE mimetic peptide (^131^EELRVRLASHLRKLRKLRLL^150^; full bar), or in presence of 1 μM Hpt and different amounts of ApoE mimetic peptide (4 or 8 μM, full bars), or in presence of 1 μM Hpt and mouse anti-human Hpt IgG (7 μg/ml, gray bar). After incubation, the cells were lysed for measurement of their radioactivity and protein concentration. The amount of cholesterol internalized was measured as dpm per mg of cell protein. Significant differences from control are indicated (^**^p < 0.001; ^***^p < 0.0001). The samples were analyzed in triplicate, and the data are expressed as means ± s.e.m.

[^3^H]-cholesterol was used as tracer. As shown in Figure 4A, Hpt significantly inhibited the cholesterol uptake mediated by ApoE at any assayed concentration (*p* < 0.005; Hpt/ApoE molar ratios in the mixtures = 25:1, 50: 1, 100:1). In particular, 0.25 μM Hpt reduced cholesterol internalization of about 15% (*p* < 0.04), while 0.5 and 1 μM Hpt reduced the internalization of 45 and 50% (*p* < 0.002). These results indicate that Hpt, due to its binding to ApoE, impaired the apolipoprotein in promoting cholesterol uptake by neurons. The specificity of this effect was confirmed by the finding that cholesterol internalization was fully restored when internalization assays were performed in the presence of both Hb and Hpt (Hb/Hpt molar ratio = 2:1). Indeed Hb is a high affinity ligand of Hpt (Bowman and Kurosky, 1982; Polticelli et al., 2008), and displaces Hpt from ApoE (Cigliano et al., 2009), which is therefore free to interact with cell receptors. Incubation of proteoliposomes with Hb alone in the culture medium did not affect cholesterol delivery to the cells. Uptake inhibition was not observed when albumin, instead of Hpt, was present in the culture medium.

Cholesterol internalization assays were also performed in the presence of both Hpt and a synthetic peptide mimicking the ApoE sequence involved in the binding to Hpt, in order to verify whether this peptide is able to rescue cholesterol uptake by neurons. As shown in Figure 4B, cholesterol internalization was reduced of 43% (*p* < 0.001) by 0.5 μM Hpt, and it was fully restored in the presence of 4 μM peptide (peptide/Hpt molar ratio 8:1). Further, cholesterol internalization was reduced of 54% (*p* < 0.0001) by 1 μM Hpt, and it was restored to 78% of the control (*p* < 0.001) in the presence of 4 μM peptide (peptide/Hpt molar ratio 4:1), and to 97% of the control (*p* < 0.0001) in the presence of 8 μM peptide (peptide/Hpt molar ratio 8:1). These results demonstrate that the ApoE mimetic peptide, as impairing ApoE interaction with Hpt, works as Hpt antagonist, rescuing the functional deficiency in cholesterol uptake, caused by Hpt. In a further control, the internalization assay was carried out in the presence of both Hpt (1 μM) and a monoclonal anti-human Hpt antibody (7 μg/ml). In these conditions cholesterol uptake was restored to 90% of the control (Figure 4B; *p* < 0.0001). These results confirm that the impairment of cholesterol uptake specifically depends on the Hpt binding to ApoE.

### Hpt influence on cholesterol-mediated toxicity

It is well known that cholesterol may be toxic to its host cell, and when accumulated, it causes cell death (Liu et al., 2010). Since ApoE mediates cholesterol internalization by neurons, we then investigated whether Hpt, by binding ApoE, was able to modulate cholesterol neurotoxicity.

**Figure 5:**
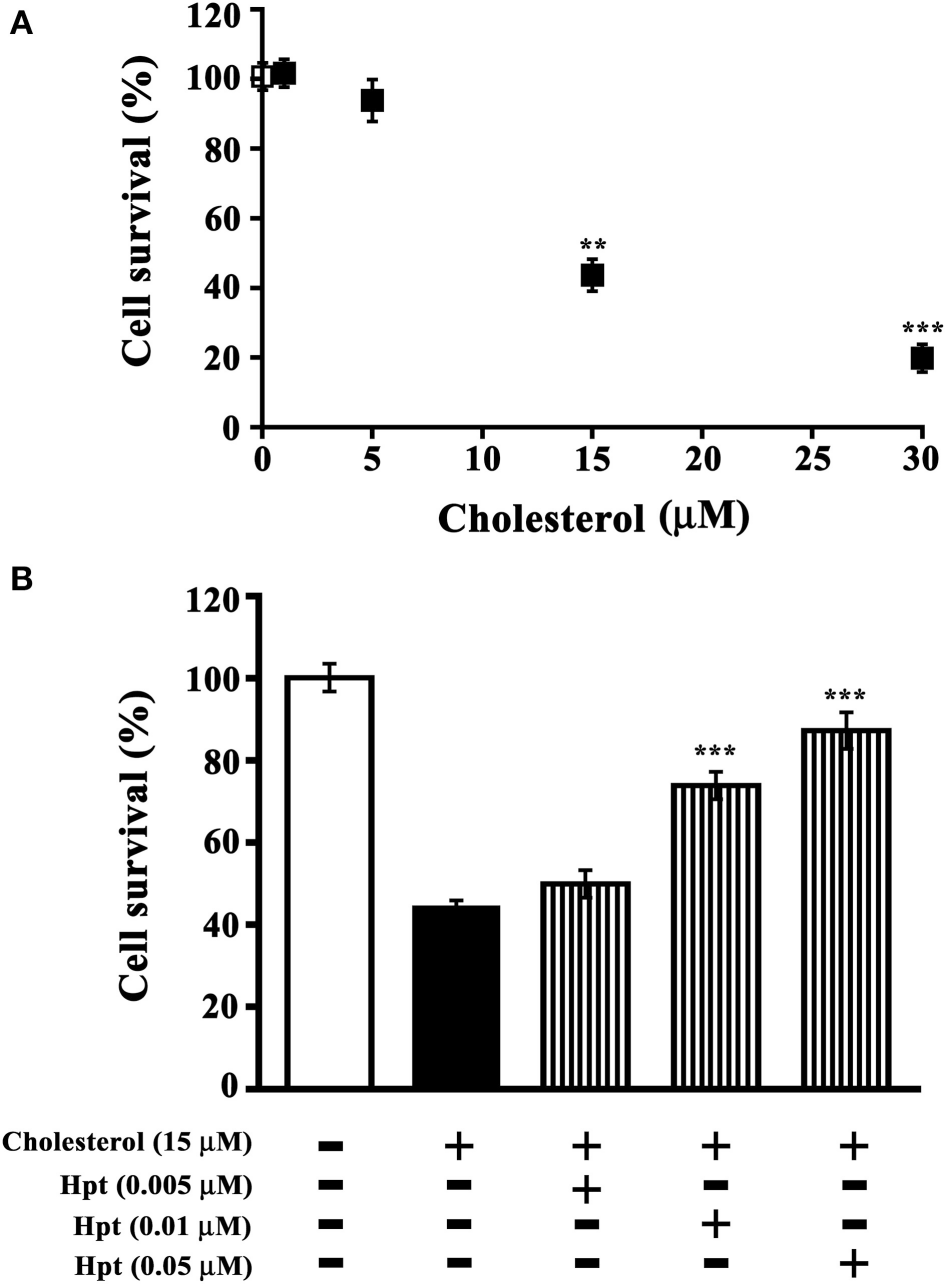
**Effect of Hpt on cholesterol-mediated neurotoxicity. (A)** Differentiated SHSY5Y were incubated into 96-well plate (20 h; 8 × 10^3^ cells per well) in DMEM containing 0.2% BSA, 10 nM ApoE, and different amounts of liposome-embedded cholesterol (0–30 μM). Cell survival was evaluated by MTT assay, and it was expressed as percentage of viability of the control (cells cultured without cholesterol addition; open square). Significant differences from control are indicated (^**^p < 0.005; ^***^p < 0.001). Data are reported as mean ± s.e.m. **(B)** Differentiated SH-SY5Y were incubated into 96-well plate (20 h; 8 × 10^3^ cells per well) in DMEM containing 0.2% BSA, 10 nM ApoE, 15 μM liposome-embedded cholesterol, and different amounts of Hpt (0.005, 0.01, or 0.05 μM; bars with vertical line). Cell survival was evaluated by MTT assay, and it was expressed as percentage of viability of the control (cells cultured without cholesterol and Hpt; open bar). Significant differences from cells treated by cholesterol (full bar) are indicated (^***^p < 0.001). Data are reported as mean ± s.e.m.

Toxic cholesterol concentrations for differentiated SH-SY5Y were determined in a preliminary experiment, by incubating cells with different amounts of cholesterol (0–30 μM) in the presence of ApoE (10 nM final concentration). As shown in Figure 5A, cholesterol treatment decreased the cell viability of differentiated SH-SY5Y in a dose-dependent manner. In particular, the treatment with 15 or 30 μM cholesterol reduced cell viability to 43.6% (*p* = 0.004) or 19.8% (*p* = 0.0001) respectively. Data were analyzed by non-linear regression and the EC50 value was 26.7 μM. The cholesterol concentration 15 μM was therefore chosen for further experiments, carried out in the presence of Hpt.

Differentiated SH-SY5Y were incubated with 15 μM cholesterol and different amounts of Hpt (0–0.05 μM), in the presence of 10 nM ApoE. As shown in Figure 5B, only 44% of cells survived after treatment with 15 μM cholesterol, and cell viability was found significantly higher (*p* < 0.001) when incubation with cholesterol was performed in presence of Hpt. In particular, cell survival was restored to 75% of the control (*p* < 0.001) by 0.01 μM Hpt, and to 90% of the control (*p* < 0.001) when treatment was carried out in the presence of 0.05 μM Hpt. These data confirm that Hpt, by binding ApoE, influences the ApoE-mediated cholesterol internalization into neuron cell lines, and suggest that Hpt might play a neuroprotective effect when cells are exposed to toxic amounts of cholesterol.

### Analysis of Hpt and ApoE in hippocampus and cerebral cortex of rats

**Figure 6:**
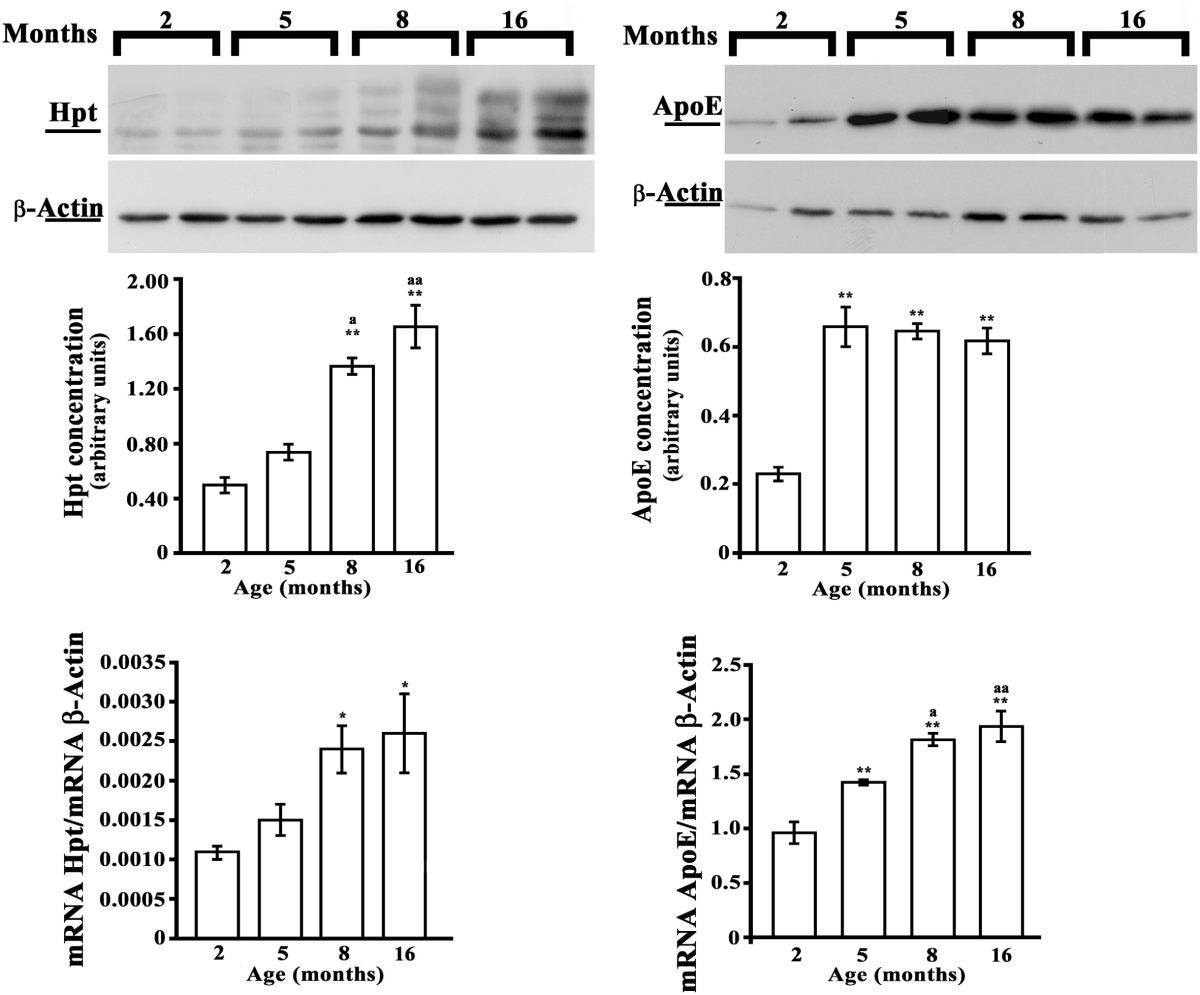
**Hpt and ApoE expression in rat hippocampus**. Hpt (left side) and ApoE (right side) protein and mRNA levels in hippocampus of 2, 5, 8, and 16 month-old rats are shown. Representative Western blots stained with anti-Hpt (left) or anti-ApoE (right) IgG, and anti-actin IgG are shown. Samples were analyzed by 12.5% SDS-PAGE and western blotting. Immunocomplexes were detected by rabbit anti-Hpt and GAR-HRP IgGs (on the left) or by goat anti-ApoE and RAG-HRP IgGs (on the right). After Hpt or ApoE detection, the membrane was stripped for reprobing with anti-β-actin. Quantitative densitometry of ApoE, Hpt, and β-actin was carried out and band intensities were calculated. Hpt and ApoE concentrations are shown relative to β-actin level. mRNA level of Hpt and ApoE are shown relative to the β-actin mRNA. The data represent the mean ± s.e.m. (eight rats per group). Significance of differences is shown. ^*^p < 0.05 vs. 2 months; ^**^p < 0.001 vs. 2 months. ^a^*p* < 0.05 vs. 5 months; ^aa^*p* < 0.001 vs. 5 months.

**Figure 7:**
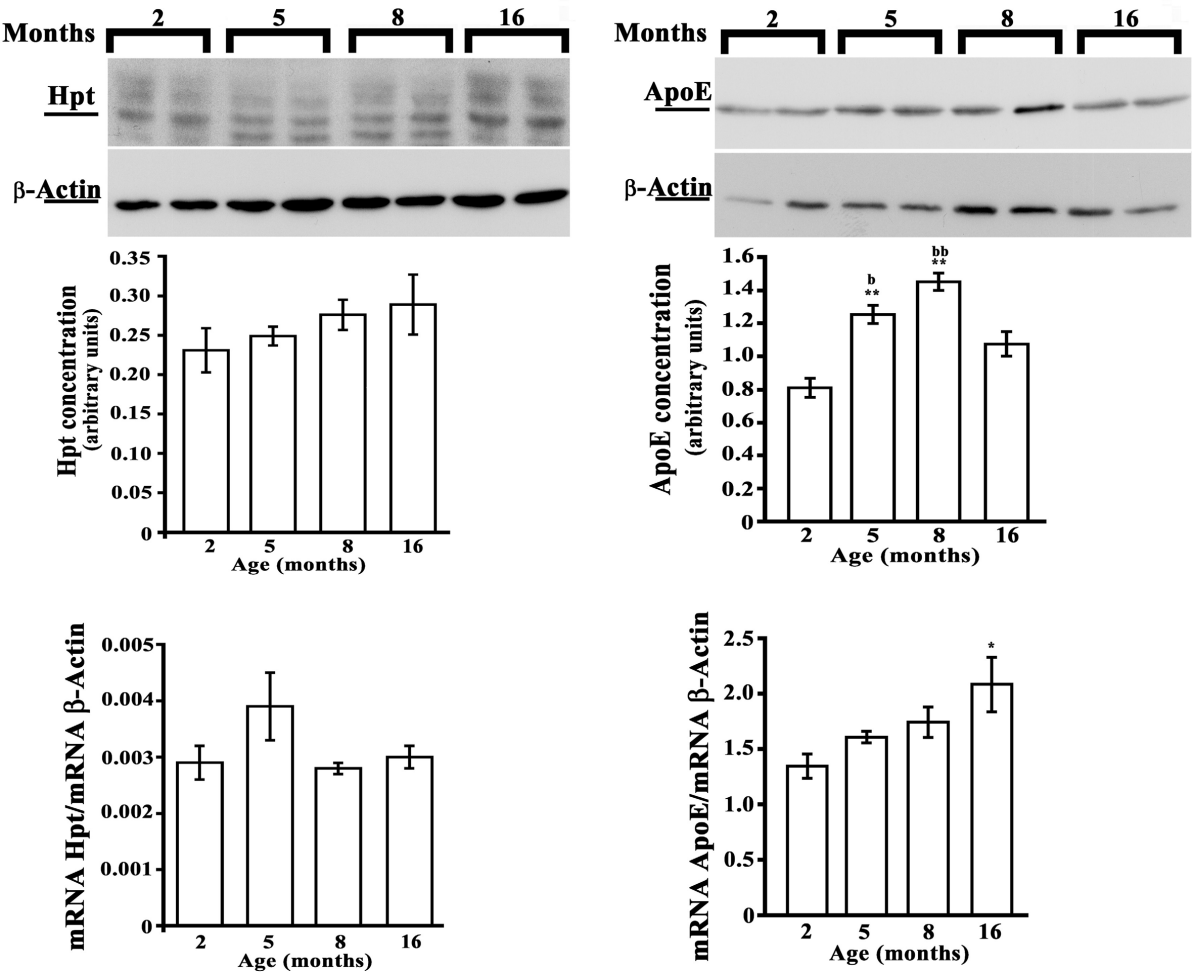
**Hpt and ApoE expression in rat cerebral cortex**. Hpt (left side) and ApoE (right side) protein and mRNA levels in cortex of 2, 5, 8, and 16 month-old rats are shown. Representative Western blots stained with anti-Hpt (left) or anti-ApoE (right) IgG, and anti-actin IgG are shown. Samples were analyzed by 12.5% SDS-PAGE and western blotting. Immunocomplexes were detected by rabbit anti-Hpt and GAR-HRP IgGs (on the left) or by goat anti-ApoE and RAG-HRP IgGs (on the right). After Hpt or ApoE detection, the membrane was stripped for reprobing with anti-β-actin. Quantitative densitometry of ApoE, Hpt, and β-actin was carried out and band intensities were calculated. Hpt and ApoE concentrations are shown relative to β-actin level. mRNA level of Hpt and ApoE are shown relative to the β-actin mRNA. The data represent the mean ± s.e.m. (eight rats per group). Significance of differences is shown. ^*^p < 0.05 vs. 2 months; ^**^p < 0.001 vs. 2 months. ^b^*p* < 0.05 vs. 16 months; ^bb^*p* < 0.001 vs. 16 months.

As no previous data are available on the effect of aging on brain expression of Hpt, we evaluated Hpt and ApoE (mRNA and protein levels) in hippocampus and in cortex of adolescent (2 month-old), adult (5 and 8 months-old), and middle-aged (16 month-old) rats. Real Time-PCR revealed a significant age-related increase of ApoE expression, which was different between the two brain regions (Figures 6, 7). In particular, the level of ApoE mRNA linearly increased with the age from 2 to 8 months in the hippocampus (Figure 6, *p* = 0.03, *r* = 0.99), while it remained unchanged in the cortex (Figure 7) until 8 months, with a significant increase in the cortex of rats of 16 months compared to the rats of 2 months (*p* < 0.05). Western blotting analysis revealed that ApoE level in the hippocampus, increased (about 2.9 fold; *p* < 0.001) in 5 month-old rats compared to 2 months, but did not further change with aging (Figure 6). In the cortex, ApoE concentration increased in 5 and 8 month-old rats compared to 2 month-old rats (1.6 and 1.8 fold respectively, *p* < 0.001), while a mild reduction was detected in 16-month-old rats when compared to 5 month-old (about 1.2 fold, *p* < 0.05) and 8 month-old (about 1.4 fold, *p* < 0.01; Figure 7). The apparent discrepancy between mRNA and protein level was observed for many proteins in mammalian cells and could be due, at least in part, to the age-related modifications in translation and/or post translation processes (Nelson and Keller, 2007). Differences in the age-dependent changes of ApoE, between hippocampus and cortex, might be due to differences in cellular composition of the two cerebral compartments. The analysis of Hpt expression in hippocampus revealed that mRNA level was significantly lower in 2 month- than in 8 and 16 month-old rats (*p* < 0.05), while no differences were found among the other groups (Figure 6). Interestingly, Hpt concentration, as detected by Western blotting, significantly increased with the age till 8 months. As matter of fact, Hpt level in hippocampus of 2 month-old rats did not differ from that of 5 months, but was 2.8 fold lower than that of 8 month-old (*p* < 0.001, Figure 6) and 3.3 fold lower than that of 16 month-old rats (*p* < 0.001; Figure 6). Further, Hpt concentration was about 2-fold higher in hippocampus of 8 month-old rats compared to 5 month-old (*p* < 0.05). Conversely, no age related changes of Hpt levels and mRNA were found in the cortex (Figure 7). The finding of Hpt mRNA in hippocampus and cerebral cortex confirms a local synthesis of Hpt in brain, which is independent from the level of the protein in plasma. Altogether these results suggest that aging is associated with changes, particularly in the hippocampus, in the Hpt/ApoE ratio.

### Analysis of Hpt and ApoE in human CSF

**Figure 8:**
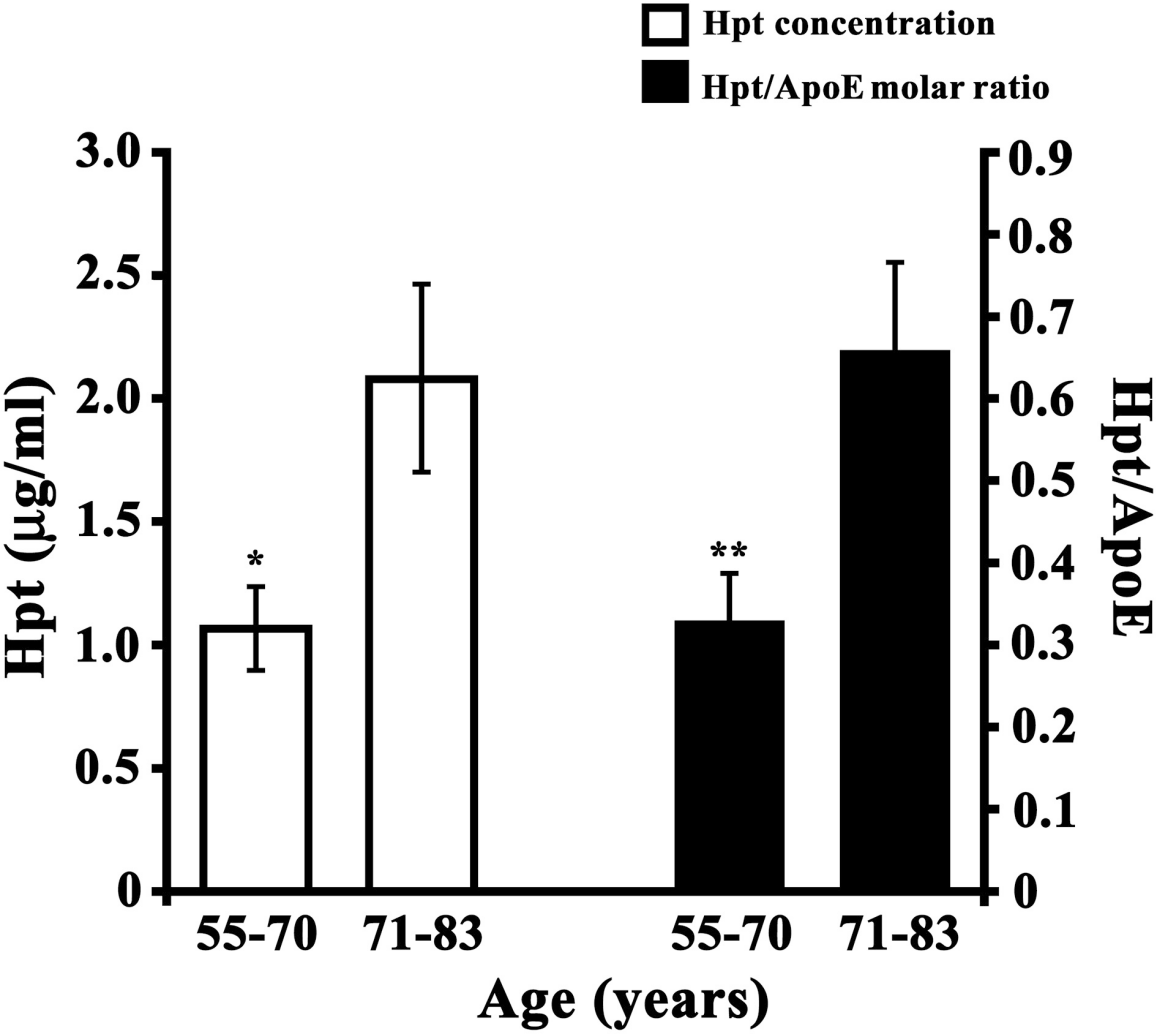
**Hpt concentration and Hpt/ApoE molar ratio in human CSF**. Hpt and ApoE concentrations were measured, by ELISA, in human CSF samples obtained from younger subjects (*N* = 13; aged 55–70 years) and older subjects (*N* = 12, aged 71–83 years). Each sample was analyzed in triplicate. Data are reported as mean ± s.e.m. Significant differences between groups are indicated (^*^p = 0.02; ^**^p = 0.01).

Hpt and ApoE concentrations were measured by ELISA in CSF samples collected from healthy subjects with age between 55 and 83 years. In detail, the samples were divided into two different groups including younger subjects (55–70 years) and older subjects (71–83 years). We found that Hpt concentration was significantly lower (*p* = 0.02) in CSF from younger donors than in CSF from older donors (1.067 ± 0.170 μg/ml vs. 2.080 ± 0.405 μg/ml; Figure 8), while ApoE level did not differ between the two age groups (2.148 ± 0.307 μg/ml vs. 2.490 ± 0.299 μg/ml). Hpt/ApoE molar ratio was lower in CSF from younger than older subjects (0.327 ± 0.060 vs. 0.656 ± 0.110 μg/ml; *p* = 0.01; Figure 8). As CSF is the biological fluid representative of the brain microenvironment, these results suggest that age-related changes of Hpt concentration occur in human central nervous system.

## Discussion

Aging is the dominant non-genetic risk factor for AD (Cummings and Cole, 2002), as it contributes to the physiological decline, and is associated, in the brain, with increased oxidative damage, inflammation, protein aggregate accumulation, demyelination, and very selective and modest neuron and synapse loss (Lee et al., 2000; Bartzokis et al., 2007). However, AD development also depends on multiple genetic and environmental risk factors. The E4 allele of ApoE represents by far the strongest and best established genetic risk factors for AD (Liu et al., 2013). ApoE4 isoform was reported to contribute to AD pathogenesis through both Aβ-dependent and Aβ-independent pathways (Liu et al., 2013). As ApoE plays a key role in modulating brain cholesterol trafficking, and dysregulation of cholesterol balance in brain is increasingly being correlated to AD (Vance, 2012), the evaluation of inflammatory- and non-inflammatory players, able to influence ApoE key activity in cholesterol or Aβ metabolism might represent an alternative approach to elucidate the link among AD pathogenesis, ApoE, and cholesterol metabolism. We previously reported that Hpt binds ApoE and affects both ApoE stimulation of lecithin:cholesterol acyltransferase and cholesterol delivery to hepatocytes (Cigliano et al., 2009). Here we report that the binding between Hpt and ApoE does not influence ApoE-dependent cholesterol efflux from astrocytes, which occurs through ABC transporters, but impairs the cholesterol delivery, mediated by ApoE, from reconstituted lipoproteins to neuron cell lines. These results suggest that Hpt binding to ApoE does not interfere with ApoE binding to ABC transporters, but might limit ApoE interaction with cell receptors. This is in agreement with our previous finding that Hpt binds the ApoE sequence ^131^EELRVRLASHLRKLRKLRLL^150^ localized in the helix 4 of the N-terminal region of the apolipoprotein (Salvatore et al., 2009). As this sequence also contains the receptor-binding region (residues 136–150) (Guttman et al., 2010; Liu et al., 2013), it is conceivable that Hpt masks by steric hindrance the ApoE domain involved in the interaction with the receptors, thus reducing cholesterol internalization by neurons. Accordingly, we found that the inhibitory effect of Hpt was reduced or abolished in the presence of a synthetic peptide that mimics the ApoE sequence involved in the binding to Hpt, as well as in the presence of the higher affinity ligand of Hpt, that is Hb (Bowman and Kurosky, 1982; Polticelli et al., 2008). In this frame, it is worth mentioning that cholesterol, even though modulating cellular metabolism can be also toxic to its host cell, and when accumulated, it causes cell death (Liu et al., 2010). Our results from *in vitro* investigations demonstrate that Hpt, at concentration similar to those measured in human CSF, plays a neuroprotective effect, reducing the toxic effect on neurons of increasing concentrations of cholesterol. Beneficial effects of Hpt were already reported, since we previously demonstrated that it protects ApoE from oxidative damage (Salvatore et al., 2009). In addition, this glycoprotein was shown to protect neuroblastoma cells from Aβ toxicity and promote the peptide uptake in macrophage-like cells (Yerbury and Wilson, 2010). Also, it plays an important role in defending neurons from damage, by neutralizing iron-rich Hb released into the brain parenchyma, after intracerebral hemorrhage (Zhao et al., 2009). Our results suggest a potential role of Hpt in modulation of brain cholesterol in addition to that played in circulation. In fact, we previously reported that Hpt influences the reverse cholesterol transport in blood, in both healthy subjects and rheumatoid arthritis patients (Cigliano et al., 2005), as well as in a mouse model of inflammation (Bucci et al., 2012).

Age-related variations of Hpt and ApoE might affect cholesterol metabolism and cell survival in brain.

Therefore we investigated whether changes of both proteins occur with aging in rats' hippocampus and cerebral cortex. ApoE concentration, in brain, was previously reported to be affected by age (Perovic et al., 2009), and our data are consistent with these results. A recent microarray analysis on hippocampal tissue showed that Hpt was between the selective transcripts induced by injections into mice hippocampus of a cocktail of tumor necrosis factor TNF-α, interleukin (IL)-12, and IL-1b (Lee et al., 2013). Interestingly, we here show, for the first time, that Hpt protein and mRNA level significantly change during aging in rat hippocampus. In particular, Hpt concentration in hippocampus significantly increases from adolescence to middle-age, while ApoE level was lower in adolescent rats than in all other groups, but did not change from adulthood to middle-age. The increase of Hpt/ApoE ratio during aging, in this brain compartment, might affect the neurons ability to recognize ApoE-containing lipoproteins, thus influencing the concentration of brain circulating lipids and modulating neurons uptake of cholesterol. Age-related changes of Hpt concentration were also found in human CSF obtained from subjects of different ages. Indeed, Hpt level was raised 2-fold in subjects aged 71-83 years with respect to subjects aged 55–70 years thus suggesting that aging is associated with changes of this protein also in human brain. Since cholesterol metabolism in the different brain regions is not uniform and, during normal aging, brain levels of this lipid differentially change depending on the region considered and cell-type studied (Martin et al., 2010), any age-related change in brain Hpt expression could have important implications. The increase of Hpt level might have deep impact in brain pathophysiology, modulating cholesterol internalization and protecting cells from cholesterol toxicity or oxidative stress, by ApoE-dependent and/or ApoE-independent mechanisms. Hpt might thus be a protective factor for ApoE function or a proinflammatory culprit during neuroinflammation. In fact, it cannot be excluded that enhanced Hpt levels, by limiting cholesterol uptake by neurons, might represent a further way by which inflammation worsens the onset and the rate of progression of neurodegeneration. We cannot assess, to date, whether the positive effects of Hpt in brain outweigh the negative effects, or vice versa. How exactly Hpt and ApoE conspire to influence neuronal functions and survival remains to be determined. However, our findings provide, for the first time, insight into the modulation of Hpt expression in aging brain, and lay the groundwork for future studies on Hpt role in brain.

## Author contributions

Maria Stefania Spagnuolo, Luisa Cigliano conceived and designed the research, performed cell biology and WB experiments, analyzed and interpreted all results, wrote the manuscript. Bernardetta Maresca, Paolo Abrescia contributed to the planning of *in vitro* experiments, performed cell biology and WB experiments, analyzed data, critically revised the manuscript. Maria Pina Mollica, Gina Cavaliere, Carolina Cefaliello, Giovanna Trinchese, Marianna Crispino contributed to the planning and execution of experiments with aging rats, analyzed data and critically revised the manuscript. Maria Grazia Esposito, Rosaria Scudiero contributed to the planning and execution of Real Time PCR experiments, analyzed results and critically revised the manuscript. All authors approved the final version to be published, and ensured that questions related to the accuracy or integrity of any part of the work are appropriately investigated and resolved.

### Conflict of interest statement

The authors declare that the research was conducted in the absence of any commercial or financial relationships that could be construed as a potential conflict of interest.
